# Multimorbidity and health-related quality of life in the older population: results from the German KORA-Age study

**DOI:** 10.1186/1477-7525-9-53

**Published:** 2011-07-18

**Authors:** Matthias Hunger, Barbara Thorand, Michaela Schunk, Angela Döring, Petra Menn, Annette Peters, Rolf Holle

**Affiliations:** 1 Helmholtz Zentrum München, German Research Center for Environmental Health (GmbH), Institute of Health Economics and Health Care Management, Ingolstädter Landstr. 1, 85764 Neuherberg, Germany; 2 Helmholtz Zentrum München, German Research Center for Environmental Health (GmbH), Institute of Epidemiology II, Ingolstädter Landstr. 1, 85764 Neuherberg, Germany

## Abstract

**Background:**

Multimorbidity in the older population is well acknowledged to negatively affect health-related quality of life (HRQL). Several studies have examined the independent effects of single diseases; however, little research has focused on interaction between diseases. The purpose of this study was to assess the impact of six self-reported major conditions and their combinations on HRQL measured by the EQ-5D.

**Methods:**

The EQ-5D was administered in the population-based KORA-Age study of 4,565 Germans aged 65 years or older. A generalised additive regression model was used to assess the effects of chronic conditions on HRQL and to account for the nonlinear associations with age and body mass index (BMI). Disease interactions were identified by a forward variable selection method.

**Results:**

The conditions with the greatest negative impact on the EQ-5D index were the history of a stroke (regression coefficient -11.3, p < 0.0001) and chronic bronchitis (regression coefficient -8.1, p < 0.0001). Patients with both diabetes and coronary disorders showed more impaired HRQL than could be expected from their separate effects (coefficient of interaction term -8.1, p < 0.0001). A synergistic effect on HRQL was also found for the combination of coronary disorders and stroke. The effect of BMI on the mean EQ-5D index was inverse U-shaped with a maximum at around 24.8 kg/m^2^.

**Conclusions:**

There are important interactions between coronary problems, diabetes mellitus, and the history of a stroke that negatively affect HRQL in the older German population. Not only high but also low BMI is associated with impairments in health status.

## Background

Multimorbidity, defined as the coexistence of two or more chronic conditions, is a common phenomenon among the older population worldwide: two recent population-based studies indicated that the prevalence of multimorbidity ranges between 40% and 56% in the general population aged 65 years and older [1,2]. Multimorbidity is known to negatively affect health outcomes including mortality, hospitalisation, and readmission [3].

Health-related quality of life (HRQL) is a health outcome measure which is increasingly used to assess the medical effectiveness of interventions and to support allocation decisions in the health care sector. Generic HRQL instruments like the EQ-5D are appropriate for non-disease-specific analyses and allow comparisons between patient groups with different medical conditions [4].

Several studies examined the effect of multimorbidity on HRQL [5-13], however research has poorly represented combinations of chronic conditions [6]. In particular, to the best of our knowledge, all studies using the EQ-5D as a measure of HRQL only considered independent disease effects [6,7,10-12]. Therefore, it has been argued that further studies should focus on identifying interaction effects between chronic conditions and account for the impact of specific disease combinations [5,7]. Synergistic effects on quality of life measures other than the EQ-5D have been reported mainly for the combination of diabetes and cardiovascular problems [8,9,14,15], the combination of respiratory and cardiovascular problems [5,9], and the simultaneous presence of diabetes and hypertension [14,16].

Studies have shown that HRQL is strongly associated with body mass index (BMI) even after adjusting for comorbidities [7,17-20]. Many contributions analysing the relationship between BMI and HRQL incorporated the effect of BMI either as a linear term, or divided its distribution in categories according to the classification of the World Health Organisation (WHO) [21]. The first approach, however, ignores the fact that association between BMI and HRQL is usually nonlinear [13,19,20,22], while in the second approach, grouping different BMI values into the same category may obscure meaningful differences within categories. Both approaches can bias findings since they may conceal the true functional form of the relationship. Furthermore, concerns have been raised about whether the WHO classification is appropriate for use in the older population [23]. In particular, the question of to what extent not only being overweight but also being underweight is associated with reduced HRQL has not been sufficiently investigated in the older population.

The purpose of this study was to clarify how different chronic conditions and pairs of conditions are associated with impairments in HRQL measured by the EQ-5D in a German general population sample of individuals aged 65 years and older. Specifically, we sought to identify and explore disease combinations which are related to HRQL over and above the independent contributions of each condition. Furthermore, we wanted to appropriately account for the association of age and BMI with HRQL by using nonparametric regression methods, i.e. without imposing a priori constraints on the functional form of this relationship.

## Methods

### Data source

The data used for analysis come from the KORA-Age study, a population-based, longitudinal study focusing on the research of long-term determinants and consequences of multimorbidity. The study design was based on the ongoing studies from the KORA research, a platform for population-based surveys and subsequent follow-up studies in the fields of epidemiology, health economics, and health care research in Germany [24]. The KORA-Age study is a follow-up of all participants aged 65-94 of the MONICA/KORA surveys S1 to S4. Participants were randomly selected from population registries from the study region, comprising the city of Augsburg and its two surrounding counties in Southern Germany. The four cross-sectional surveys were conducted between 1984 and 2001, and participation rates ranged between 79% in S1 and 67% in S4 [25]. Details about study design, sampling method and data collection can be found elsewhere [24-26]. In total, 17,607 people participated in at least one of the four surveys. The KORA-Age study population is restricted to the subgroup of 9,197 subjects who were born in 1943 or earlier. 2,734 of these 9,197 individuals had died, 45 moved abroad or to an unknown location, and 427 refused to be contacted for any follow-up. A follow-up questionnaire for self-completion with questions on chronic conditions and the EQ-5D was posted to the remaining 5,991 eligible people with known addresses between November 2008 and September 2009. All recipients who did not answer within 4 weeks were sent a postcard reminder. After another 4 weeks, non-respondents were contacted by telephone and if the person could be motivated to participate, the questionnaire was administered via telephone.

In total, data was collected for 4,565 people (response 76.2%), of whom 3,833 returned the questionnaire and 732 (16.0%) were interviewed via telephone.

The KORA-Age study was approved by the Ethics Committee of the Bavarian Medical Association.

### Chronic conditions and sociodemographics

Information on chronic conditions was based on self-reports. The history of stroke was ascertained by asking the questions: "Have you ever had a stroke diagnosed by a physician?" and "If yes, how many strokes have you had?" Furthermore, for each stroke, respondents were asked to report the year of diagnosis. History of myocardial infarction and history of cancer diagnosis were assessed in the same manner. History of bypass operation was assessed similarly, but only the year of the first operation had to be reported. To identify subjects with diabetes and hypertension, participants were asked if they had ever been told by a physician that they had each condition. Following the definition from the WHO, participants were identified as suffering from chronic bronchitis if they reported having a cough and sputum during most days in three consecutive months (two questions).

Body weight was self-reported whereas information on body height was obtained from measurements performed at the baseline examinations. From these data, BMI was calculated as weight in kg divided by the squared height in meters. Age and a three-level categorical education variable (primary, secondary and tertiary education) were also taken from the baseline surveys.

In our regression analyses, all chronic conditions were considered as binary covariates. Subjects with a myocardial infarction and/or a bypass operation were classified as having coronary disorders [7].

### EQ-5D

The EQ-5D is a generic measure of HRQL which can be used for describing and valuing health states. It consists of a self-reported health state description and a visual analogue scale (VAS). The self-reported description comprises five questions referring to the dimensions mobility, self-care, usual activities, pain/discomfort and anxiety/depression. Each dimension has three response levels (no, moderate, or extreme problems), generating a total of 243 different health states. These health states can be transformed into a single utility value (EQ-5D index) using a scoring algorithm which is based on valuations of representative general population samples. This study used the European tariff suggested by Greiner et al. [4]. Due to lack of space, our questionnaire did not include the VAS; however, the EQ-5D index is calculated independently of the VAS.

### Statistical analyses

We conducted multiple regression analyses for the EQ-5D index to determine the simultaneous effect that chronic conditions and demographic variables have on HRQL in our sample. To account for potential nonlinear associations of age and BMI with the EQ-5D index, we fitted additive models which are special cases of generalised additive models (GAMs) where the error terms are assumed to follow a normal distribution [27]. Our model can be written as

where *Y_i _*is the response of individual *i*, *f_age _*and *f_BMI _*are smooth functions, and **x**_i_^T^**β **is a linear predictor including the binary and categorical covariates of the model. As in the linear model, all *ε_i _*are independently distributed zero-mean Gaussian variables with variance σ^2^.

The smooth functions *f_age _*and *f_BMI _*were estimated by using thin plate regression splines, and smoothing parameters were estimated through generalised cross-validation (GCV) [27]. In the additive model, the effects of the binary and categorical covariates (i.e., chronic conditions or sociodemographic variables) are interpreted as in the linear model while we represent the effect that age and BMI exert on HRQL by plotting the estimated smooth functions  and . We checked robustness of the estimated curves by splitting the data into two age- and BMI-matched subsets and refitting the regression model.

First, we fitted a regression model where all available covariates were included as main effects. We also included a binary covariate to distinguish between respondents who returned the questionnaire and those who were interviewed by telephone. Second, we assessed the effects of disease combinations by adding to the model the significant pairwise interaction terms between diseases. To identify the significant interactions, we used a stepwise forward selection procedure. In each cycle, the interaction term with the smallest p-value (based on the corresponding F-test) was included until all disease interactions in the model were significant at the 1% level [27,28]. We decided to use a significance level of 1% in order to obtain more stable results: since we considered 15 pairs of diseases, approximately one interaction term would be expected to be significant at the 5% level by chance. This effect is lessened if one uses a stricter significance level.

To assess the sensitivity of the results to missing data, we refitted the models to an imputed data set. We used the Markov Chain Monte Carlo method to impute missing covariates and the predictive mean matching method to impute missing values in the EQ-5D index [29]. We performed single instead of multiple imputation due to the relatively low percentage of missing values and the high computational effort that is associated with analysing additive models based on multiply imputed datasets.

Studies have found that the same condition may have a different impact across age and sex groups [11]. To examine possible interaction effects between age and specific chronic diseases, we split the data into a subset of younger (65 - 74 years) and a subset of older (> = 75 years) participants. We refitted the model to these two subsets and compared the estimated regression coefficients. In the same way, we examined interaction effects with sex.

The regression model for the EQ-5D index scores determines the independent effects that chronic conditions have on overall health. However, as the EQ-5D index is a weighted summary score of five items representing different dimensions of health, decreases in the EQ-5D index score may arise from different patterns of impairments across these individual dimensions. To examine how the chronic conditions in question affected the EQ-5D health dimensions, we additionally fitted logistic generalised additive models for each EQ-5D item, merging response categories 2 ('moderate problems') and 3 ('severe problems') into one category to form a dichotomous dependent variable. To be consistent with the EQ-5D index model, we report the results of both main effects and interaction models, which include the same disease interactions as the EQ-5D index model. In particular, this approach allows us to investigate whether the interactions between diseases found in the EQ-5D index model can mainly be ascribed to specific dimensions of health.

Data analyses were carried out using the statistical software R with the add-on package mgcv [27,30]. Missing value imputation was performed using SAS 9.1 (SAS Institute, Cary, North Carolina, USA).

## Results

Sample characteristics are presented in Table 1 for the whole sample as well as stratified by the data collection method (questionnaire vs. telephone respondents). From the 4,565 patients in the whole sample, 2,198 (48.1%) were male. Mean age was 73.9 years (SD 6.23) and the oldest respondent was 94 years old. Hypertension (59.2%) and diabetes mellitus (17.5%) were the most prevalent conditions. Respondents interviewed by telephone were more likely to be female (58.9% vs. 50.5%, p < 0.0001, chi-square test) and on average 2.6 years older (CI: 2.1 - 3.1). Furthermore, they had more chronic conditions on average than the questionnaire respondents.

**Table 1:** Socio-demographic characteristics and self-reported prevalence of chronic conditions in the study population

Variable	All respondents N = 4,565	Questionnaire respondents N = 3,833	Telephone respondents N = 732
Mean age, years (SD)	73.86 (6.23)	73.44 (6.04)	76.03 (6.70)
Male sex, n (%)	2,198 (48.1%)	1,897 (49.5%)	301 (41.1%)
Education, n (%)			
Primary	3,328 (70.7%)	2,665 (69.6%)	563 (76.9%)
Secondary	803 (17.6%)	699 (18.2%)	104 (14.2%)
Tertiary	532 (11.7%)	467 (12.2%)	65 (8.9%)
Mean BMI, kg/m^2 ^(SD)	27.45 (4.44)	27.39 (4.34)	27.76 (4.93)
Diabetes mellitus, n (%)	798 (17.5%)	652 (17.0%)	146 (20.0%)
Chronic bronchitis, n (%)	297 (6.5%)	241 (6.3%)	56 (7.7%)
Hypertension, n (%)	2,700 (59.2%)	2,240 (58.5%)	460 (63.1%)
Coronary event, n (%)	461 (10.1%)	376 (9.8%)	85 (11.6%)
Bypass operation, n (%)	178 (3.9%)	147 (3.8%)	31 (4.2%)
Myocardial infarction (MI), n (%)	395 (8.7%)	325 (8.5%)	70 (9.6%)
Mean time since last MI, years (SD)	10.49 (8.24)	10.69 (8.32)	9.52 (7.82)
Stroke, n (%)	330 (7.2%)	248 (6.5%)	82 (11.2%)
Mean time since last stroke, years (SD)	6.72 (6.57)	6.55 (6.45)	7.27 (6.95)
Cancer, n (%)	608 (13.3%)	511 (13.3%)	97 (13.3%)
Mean time since last diagnosis, years (SD)	8.79 (8.64)	8.58 (8.52)	9.94 (9.26)

SD: standard deviation

The EQ-5D index could not be calculated for 93 (2.0%) respondents due to missing values in at least one EQ-5D item. Excluding participants with missing data in the EQ-5D index or in the covariates reduced the final sample size from 4,565 to 4,412. The 153 (3.35%) individuals excluded from analyses were on average 2.9 (CI: 1.9 - 3.9) years older than the participants with complete information and were slightly more likely to be female (p = 0.016, chi-square-test).

The mean EQ-5D index in the sample was 76.3 (SD 18.8) and the observed values covered the entire range from 3.5 to 97.7. There was a ceiling effect in the data since 1,285 (29.1%) respondents stated having no problems in any of the five EQ-5D dimensions and were hence assigned the EQ-5D index value 97.7. Moderate or severe problems were most frequently reported in the EQ-5D dimension 'pain' (62.5%), followed by 'mobility' (31.3%), 'anxiety/depression' (29.2%), 'usual activities' (22.5%) and 'self-care' (10.1%).

Respondents interviewed by telephone rated their health on average 8.0 (CI: 6.2 - 9.7) points lower than the questionnaire respondents (unadjusted for covariates).

Results from the additive regression analyses are shown in Table 2. In the main effect model, all conditions were associated with significantly decreased EQ-5D index scores. The most severe impairments were observed for stroke (-11.3) and chronic bronchitis (-8.1). In the interaction model, two disease combinations with synergistic effects were observed: in the combination of diabetes mellitus and coronary disorders, both conditions alone had no effect on HRQL, but their combination was associated with significantly reduced EQ-5D scores. In the combination of coronary disorders and stroke, the history of a stroke had a negative main effect on HRQL, and the effect of coronary disorders alone was not significant. However, patients suffering from both conditions were more seriously impaired than could be expected from the independent effects. In both regression models, one observed lower mean EQ-5D scores for females and higher HRQL for respondents with tertiary education. Telephone interview respondents reported on average 4.5 points lower EQ-5D scores than questionnaire respondents.

**Table 2:** Estimated regression coefficients from the additive model

		Main effect model (adj. R^2 ^= 0.171)				Model with interaction terms (adj. R^2 ^= 0.177)			
**Covariate**		**Estimate**	**95% confidence limits**		**p-value**	**Estimate**	**95% confidence limits**		**p-value**

Intercept		81.85	80.74	82.96	< 0.0001	81.56	80.44	82.67	< 0.0001
Data collection*	Telephone interview	-4.49	-5.89	-3.08	<0.0001	-4.55	-5.95	-3.15	<0.0001
Age (years)		see Figure 1			<0.0001	not shown			<0.0001
BMI (kg/m^2^)		see Figure 1			<0.0001	not shown			<0.0001
Sex^†^	Female	-4.06	-5.13	-2.99	<0.0001	-3.96	-5.03	-2.89	<0.0001
Education^‡^	Secondary	0.07	-1.29	1.44	0.9151	0.06	-1.30	1.42	0.9332
	Tertiary	3.71	2.08	5.34	<0.0001	3.72	2.10	5.35	<0.0001
Diabetes mellitus		-2.49	-3.89	-1.10	0.0004	-1.27	-2.77	0.22	0.0946
Coronary event		-3.94	-5.67	-2.21	<0.0001	-0.64	-2.73	1.45	0.5462
Stroke		-11.25	-13.23	-9.26	<0.0001	-9.44	-11.66	-7.22	<0.0001
Cancer		-2.67	-4.16	-1.17	0.0005	-2.79	-4.28	-1.30	0.0002
Chronic bronchitis		-8.10	-10.18	-6.03	<0.0001	-8.14	-10.21	-6.07	<0.0001
Hypertension		-1.15	-2.22	-0.07	0.0367	-1.23	-2.30	-0.16	0.0248
Diabetes*coronary event						-8.12	-11.95	-4.28	<0.0001
Coronary event*stroke						-8.28	-13.17	-3.38	0.0009

The dependent variable is the EQ-5D index score.

BMI: Body mass index

*Reference category: Questionnaire respondents

†Reference category: Male sex

‡Reference category: Primary Education

All events are self-reported

**Figure 1:**
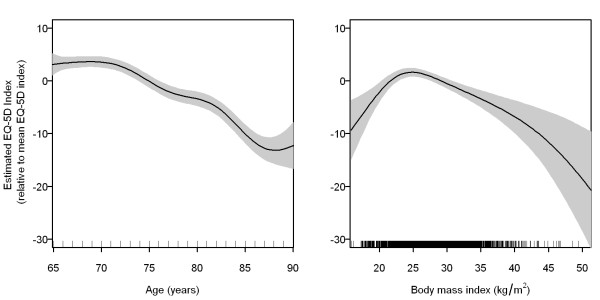
**Estimated smooth functions  and  showing the nonlinear effects of age and body mass index (BMI) on the mean EQ-5D index score**. The solid curves represent GAM estimates using a thin plate regression spline function with estimated 6.2 and 4.7 effective degrees of freedom for age and BMI, respectively. The shaded areas represent approximate 95% pointwise confidence intervals. The functions are fixed around the mean value of the EQ-5D index score. Due to estimation uncertainty for outliers, values for seven subjects over 90 years and one subject with a BMI >50 are not displayed.

The nonlinear effects of age and BMI on the mean EQ-5D index are represented by the estimated smooth functions  and  in Figure 1. Since effects were almost identical in the two regression models, we only show the curves of the main effect model. The left curve in Figure 1 expresses a nonlinear age effect with stable health until 70 years and a decline in HRQL from the age of 70. Between 70 and 85 years, HRQL decreased on average by 14 units. The right curve in Figure 1 shows that the relationship between BMI and HRQL was inverse U-shaped with the maximum HRQL located around a BMI of about 24.8 kg/m^2^. It indicates that an increase of the BMI from 24.8 to 35 was associated with an EQ-5D utility loss of about 5.0 units. On the other hand, underweight individuals with a BMI of 18 had an average impairment of 7.1 units compared to a BMI of 24.8. The estimated curves differed only slightly when the data were split into two subsets.

Applying the regression model to the imputed data set did not alter findings and the same interactions were selected. Separately fitting the regression model to the subset of younger and to the subset of older participants, we observed that the history of a stroke had a stronger negative impact on HRQL in individuals aged 65-74 years than in individuals aged 75 years and above. In contrast, the impairments in HRQL associated with the history of cancer were more pronounced in the older age group. Furthermore, the negative effect of low BMI on HRQL was more important in the older age group (results not shown).

The stratified analysis by sex revealed that the history of a stroke had a stronger effect in men (-14.3; CI: -16.9 - -11.7) than in women (-7.4; CI: -10.5 - -4.3). In contrast, the effect of chronic bronchitis was slightly more pronounced in women (-10.5; CI: -14.4 - -7.6) than in men (-6.4; CI: -9.0 - -3.7). Moreover, HRQL for men peaked at a BMI of 26.3 kg/m^2 ^while maximum HRQL in women was observed at a BMI of 23.4 kg/m^2^.

Results from the logistic regression models are shown in Table 3. The covariates considered explained less variance in the items 'pain' and 'anxiety/depression' (deviance explained 5.3% and 4.2%, respectively) than in the other items (deviance explained between 13.1% and 16.5%). The synergistic effects found in the EQ-5D index model were also reflected in the EQ-5D dimensions, however, the interaction terms were only partly significant. Sex differences were mainly observed in the dimensions 'usual activities', 'pain', and 'anxiety/depression'.

**Table 3:** Estimated odds ratios from the logistic generalised additive model

		Mobility		Self-care		Usual activities		Pain		Anxiety/Depression	
Sex^†^	female	1.16	1.15	1.14	1.13	1.35**	1.33**	1.45***	1.44***	2.13***	2.12***
Diabetes mellitus		1.49***	1.39**	1.51**	1.29	1.53***	1.33**	1.06	0.98	1.16	1.06
Coronary event		1.34*	1.11	1.57**	1.05	1.58***	1.17	1.27*	1.04	1.41**	1.18
Stroke		3.09***	2.81***	3.96***	3.44***	3.24***	3.04***	1.96***	1.82***	2.07***	2.02***
Cancer		1.35**	1.36**	1.30	1.32	1.45**	1.47***	1.07	1.08	1.22*	1.22*
Bronchitis		2.33***	2.34***	2.44***	2.47***	2.21***	2.24***	2.40***	2.41***	1.72***	1.73***
Hypertension		1.19*	1.19*	1.04	1.05	1.17	1.17	1.25*	1.26**	1.19*	1.20*
Diabetes*coronary event		-	1.56	-	2.17*	-	2.36**	-	1.99*	-	1.81*
Coronary event*stroke		-	1.69	-	1.83	-	1.37	-	1.51	-	1.09

Deviance explained		13.1%	13.2%	16.5%	16.2%	13.3%	13.6%	5.3%	5.5%	4.2%	4.3%

The dependent variables are the probability of reporting moderate or severe problems in the respective EQ-5D dimension. In each dimension, the first column refers to the main effect model and the second column to the interaction model.

All estimates are adjusted for age, body mass index, data collection method, education.

†Reference category: Male sex

* p < 0.05

** p < 0.01

*** p < 0.001

All events are self-reported

## Discussion

In our study, we found that each of the chronic conditions considered was associated with impairments in HRQL, either alone or in combination with other conditions. In agreement with the results of other studies, we observed the most severe impairments for a history of stroke [7,10,31] and chronic bronchitis [7,32]. With an adjusted R^2 ^of approximately 18%, our models only explained a moderate proportion of variance. Nevertheless, the adjusted R^2 ^was equal to or better than in comparable studies [7,12,33].

Several researchers investigated the joint effects that specific disease combinations have on quality of life. However, to the best of our knowledge, this study is the first to explicitly examine interaction effects between chronic conditions on HRQL measured by the EQ-5D. Our analyses revealed that the combination of diabetes mellitus and coronary problems, as well as the combination of coronary problems and a stroke history were synergistically associated with HRQL. There was no subtractive interaction between diseases in our data.

The joint effect of diabetes and coronary problems on HRQL in our study reflects the substantial burden of illness caused by the combination of these two conditions. Studies have shown that persons with diabetes are at greatly increased risk of cardiovascular diseases and that the prevalence of cardiovascular complications amongst persons with diabetes is especially high in older age groups [34]. Our results complement these findings by underlining the exacerbating effect that cardiovascular diseases show on HRQL in subjects with diabetes. Synergistic effects of diabetes and coronary problems on HRQL have also been reported in studies using the SF-36 [9,15] and the HUI3 [8], as well as in studies on disability and functional status [14,35]. In contrast, Wee et al. reported mainly additive, but even partly subtractive effects of heart disease and diabetes on the SF-36 subscales and the SF-6D [16]. For a discussion of further synergistic relationships found in literature, see, e.g., Hodek et al. [36].

The nonsignificant main effect of diabetes in our interaction model indicates that either there is no decline in HRQL caused by diabetes without coronary comorbidity, or that the decline is too low to be detected by the EQ-5D. It has been argued that the EQ-5D detects differences due to diabetes related complications, but that it lacks sensitivity in capturing differences between diabetes treatment regimes [37]. Although there is evidence that subjects with diabetes but without comorbidities still have more impairments than subjects without diabetes [14,15], another study found that diabetes was not associated with lower EQ-5D scores after adjusting for comorbidities [7]. Rijken et al. even observed a positive main effect of diabetes on the physical scale of the SF-36 when the negative interaction term with cardiovascular disease was accounted for [9].

We found the combination of coronary problems and the history of a stroke to also have synergistic effects on HRQL. Stroke and myocardial infarction are both mainly manifestations of atherosclerosis. Studies showed higher mortality rates and increased treatment cost when stroke occurs after myocardial infarction [38,39]. Reversely, myocardial infarction is an important cause of death in patients with cerebrovascular disease [40]. Another study found that heart disease and stroke were synergistically associated with physical disability [35]. Our results highlight the negative impact of this disease combination on HRQL.

Very few studies on HRQL in multimorbid patients accounted for the effect of weight problems, as expressed by the BMI [7,17,18,41]. And to the best of our knowledge, this study is the first to explicitly examine the functional form of the relationship between BMI and HRQL by means of semiparametric regression methods, i.e., without imposing a priori constraints on its shape such as polynomial forms or piecewise constant terms. Our analyses showed that the relationship between BMI and HRQL was inverse U-shaped and that not only overweight but also lower BMI values were associated with significantly reduced HRQL. This supports findings reported by other studies [13,19,20,22]. Furthermore, our study is the first to address the nonlinear association of BMI with HRQL in older adults. Ignoring the nonlinearity would overestimate HRQL for subjects with lower BMI values, which is particularly serious in the older population where being underweight can be a severe problem [42].

The additive regression models used in our study also allowed us to explore the nonlinear relationship between age and HRQL. In our sample, age was strongly associated with the mean EQ-5D index, but the age-related decline in health was only observed from the age of 70. The negative correlation between age and HRQL, even after adjustment for the effect of chronic conditions, is supported by several studies [6,7,10,43]. However, there is evidence that age per se is only a weak predictor of HRQL and that rather the increasing number and severity of chronic conditions are behind the age effect [7,11,36,43]. Thus, the association between age and HRQL may become less pronounced if morbidity was assessed by a greater number of comorbidities or if disease severity was accounted for.

The data used in our analyses came from a postal questionnaire for self-completion. However, about 16% of the participants were interviewed by telephone since these people had not returned the questionnaire despite being sent a reminder. Respondents interviewed by telephone were on average older, more likely to be female and suffered from more chronic conditions than the questionnaire respondents. These three aspects are all known to be negatively associated with HRQL. In fact, the unadjusted HRQL score within the telephone respondents was nearly 8 points lower than within the questionnaire respondents. However, our multivariable regression analyses showed that the difference in HRQL between the two data collection methods persisted even after adjustment for covariates. There are two possible explanations for this finding: first, it can not be ruled out that answers to quality of life questions given by the telephone respondents may be biased due to the personal interview situation [44]. Second, it is possible that the difference is caused by unobserved comorbidities. Although the telephone interviews could increase the response rate, our study (as most population surveys) was still confronted with the problem of non-response. An extensive analysis on this issue in one of the baseline surveys has shown that non-respondents included a higher percentage of people with impaired health [45], and it can be assumed that more severely ill subjects were less likely to participate in our study. As a consequence, our results may underestimate the burden of comorbidity in the older population. Nevertheless, the prevalence of the chronic conditions in our study sample was comparable to that reported in another German study with the same age range [7].

One strength of our study is the large number of patients with cell frequencies for disease combinations that allow for the valid examination of interaction terms. Also, our study is population-based so that results are more likely to be transferable to the older general population than results obtained from general practice samples. Finally, to our knowledge, our study is the first to examine the effect of disease combinations on HRQL measured by the EQ-5D, the most frequently used instrument in economic evaluation.

A limitation of our study was that we relied on a limited list of only six chronic conditions and no psychiatric condition was amongst the considered conditions [3]. This limitation is reflected by the relatively low proportions of explained deviance in the regression models, especially for the EQ-5D item 'anxiety/depression'. Also, we did not assess dementia because questions about the diagnosis of dementia are a sensitive issue and responses may be of limited validity [46]. However, most of the comparable studies evaluating interaction effects considered a similar number of chronic conditions [9,16,35], and our study considered most of the common widespread diseases in western countries.

Another limitation is that the presence of chronic conditions in our study was based on self-reports. We did not use a specific, validated questionnaire; however, the case-finding questions for physician-diagnosed illness used in our questionnaire are widely used in population-based studies [8,35,47]. Self-reports are not as valid as medical record information, however, they have been shown to provide reasonable estimates of comorbidity in the older population [48,49]. In an earlier follow-up of the KORA S1-S3 participants, the diagnoses of myocardial infarction, stroke, and diabetes have been validated by medical record review and the agreement was very high [50].

Furthermore, our analyses did not account for time since diagnosis or disease severity. Although long-term reductions in HRQL for patients with a history of myocardial infarction or stroke were reported in literature [51,52], disease burdens may be higher for more recent diagnoses. Differentiating by disease duration and disease severity would permit more precise quantification of the association between individual conditions and HRQL. However, this study focused on exploring the joint effects of disease combinations, and interaction effects between conditions could no longer be described comprehensively if the effect of each diagnosis was additionally differentiated by severity or disease duration. Finally, the effects that specific disease combinations have on HRQL may be more complex than described by pairwise interaction terms. However, three-way or even higher order interactions are complicated to interpret and their estimation is likely to be unstable in our data due to small cell frequencies of some three-way combinations. Nevertheless, the pairwise disease interactions in our study can be considered as a reasonable approximation of potentially more complex dependencies [35].

## Conclusions

The effects of chronic conditions on HRQL in the older population are not always purely additive. Our study showed that the interactions between coronary problems, diabetes mellitus, and the history of stroke caused greater impairments in HRQL measured by the EQ-5D than could be expected from the separate effects of these conditions. Our findings emphasise the importance of comorbidity prevention in order to reduce the health burden caused by the exacerbating effects of specific disease combinations.

## Competing interests

The authors declare that they have no competing interests. The KORA-Age project was financed by the German Federal Ministry of Education and Research (BMBF FKZ 01ET0713).

## Authors' contributions

MH devised the concept for the paper, performed the statistical analysis, interpreted the data and drafted the manuscript. BT was involved in the coordination of the study and commented on drafts of paper. MS was involved in the interpretation of data. AD participated in the coordination of the study. PM commented on drafts of paper. AP was involved in the conception of the study. RH was involved in the conception of the study and assisted in writing the manuscript. All authors have read and approved the final version of the manuscript.
